# Personal Growth Through Products and Brands: Michelangelo Phenomenon and Product Affirmation

**DOI:** 10.3390/bs16081398

**Published:** 2026-08-14

**Authors:** Madoka Kumashiro, Michael K. Coolsen, Keith A. Quesenberry

**Affiliations:** 1 Department of Psychology and Neuroscience, Goldsmiths, University of London, London SE14 6NW, UK; 2 Department of Management, Marketing, and Entrepreneurship, Shippensburg University, Shippensburg, PA 17257-2299, USA; mkcool@ship.edu; 3 Department of Markets, Innovation & Design, Freeman College of Mananagement, Bucknell University, One Dent Drive, Lewisburg, PA 17837, USA; kq002@bucknell.edu

**Keywords:** product affirmation, movement toward ideal self, personal growth, well-being, brand loyalty, Michelangelo phenomenon

## Abstract

This conceptual review bridges the interpersonal and consumer-behavior relationship studies to examine how consumer products can function as symbolic relationship partners that can facilitate personal growth. We apply the Michelangelo phenomenon, an interpersonal model of personal growth, to propose a model of product affirmation, in which products, services, or brands can be used by consumers to experience personal growth, much in the same manner that close relationship partners can help individuals move toward their ideal selves. The model proposes that products and brands may serve as symbolic relationship partners that may be able to affirm consumers’ desired traits, values, aspirations, and identities, which in turn can support personal growth and enhance personal well-being and brand loyalty. We review the theoretical foundations of this affirmation process by drawing parallels between the literature on interpersonal processes and consumer–brand behavior to develop a conceptual basis for this model. We also discuss potential negative consequences when brands reinforce maladaptive, unrealistic, or externally imposed ideals. We conclude with theoretical and practical implications, including for marketing practice, and directions for future research.

## 1. Introduction

The “Just Do It” slogan, introduced by the athletic gear company Nike almost four decades ago (an illustrative example from Creative Review, 2012), has endured as an iconic message with a universal appeal that anyone can overcome procrastination and hesitation about physical fitness by simply getting started. With a simple and recognizable swoosh symbol, the sporting corporation sells the message that wearing its products would help consumers become healthier and fitter. Similar themes appear across brands, promising that engaging with their products can help consumers become healthier, smarter, more professionally successful, creative, confident, authentic, attractive, and adventurous. Thus, brands often sell visions of the person consumers could become.

Such branding strategies raise an important research question: can brands facilitate personal growth? Personal growth has been described as the motivation “to develop one’s potential, to grow and expand as a person” (Ryff, 1989, p. 1071). While advertising campaigns like Nike’s may encourage people to try out sporting activities, many people may not stick with the activity, with the sporting gear consigned to the closet. The key to lasting engagement in personal growth activities may lie in whether the activity is part of one’s ideal self, or the most desired future potential self (Higgins, 1987, 1989; Markus & Nurius, 1986). Individuals are often motivated to take action to minimize the discrepancies between one’s actual self and ideal self (Higgins, 1987, 1989), and as such, brands may exert greater and more enduring influence when consumers feel closer to their ideal selves. For example, if being athletic is part of one’s ideal self, individuals may be more likely to stick to exercising, but if this is not part of one’s ideal self, individuals may find it more difficult to muster the motivation to keep exercising. Moreover, if individuals experience such personal growth with the help of a brand, they are more likely to remain loyal to the brand.

Although consumer research has shown that consumers often show a preference for products that reflect or help them symbolically feel closer to their ideal self (e.g., Sirgy, 1982; Wicklund & Gollwitzer, 1981), it is unclear if engaging with such brands repeatedly can facilitate movement toward their overall ideal self. The current conceptual review bridges this gap through mapping conceptual parallels between theories from interpersonal relationships and the consumer–brand relationship literature, to elaborate on a model of *product affirmation* (Coolsen & Kumashiro, 2009; Coolsen et al., 2010), which we mainly apply to brands for the purpose of this paper^1^. In doing so, we adopt the approach of the emerging consumer–brand relationship literature (e.g., Fournier, 1998), which shifted focus away from viewing consumer behaviors as just a transactional exchange and toward perceiving brands as a relationship partner, playing a meaningful role in individuals’ lives just as a human relationship partner might.

**Figure 1:**
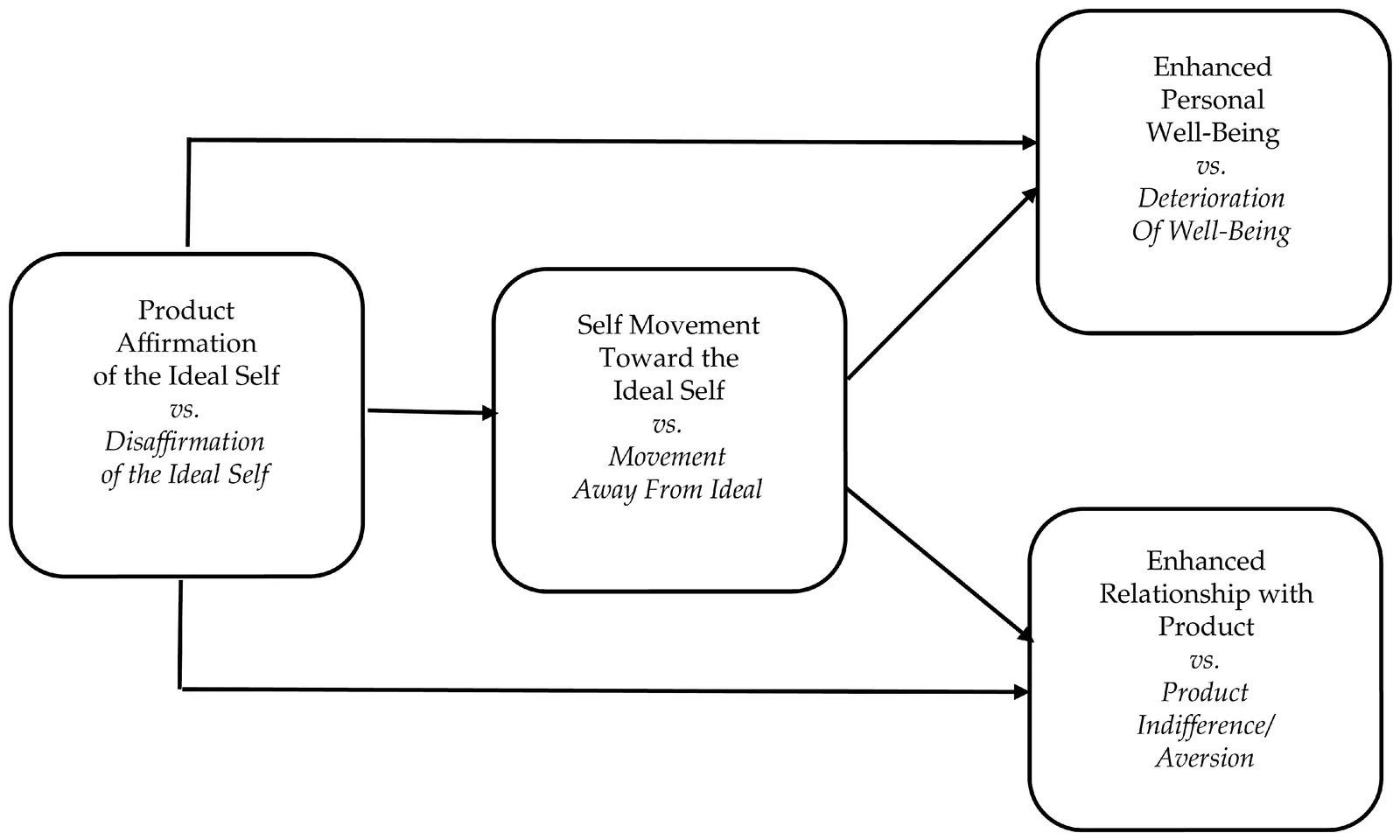
Proposed product affirmation model. Product affirmation of the ideal self is proposed to predict movement toward the ideal self. Movement toward the ideal self is hypothesized to mediate the associations between product affirmation and two proposed outcomes: enhanced personal well-being and an enhanced relationship with the product. The model also proposes possible direct associations between product affirmation and these outcomes. Conversely, product disaffirmation and movement away from the ideal self are proposed to be associated with deterioration in personal well-being and product indifference or aversion. The pathways shown represent theoretical propositions and have not yet been tested as a complete causal model in consumer contexts. The figure was adapted from the Michelangelo phenomenon (e.g., Rusbult et al., 2009a), by replacing partner affirmation with product affirmation and relationship well-being with the consumer’s relationship with the product.

Using this approach, we apply the Michelangelo phenomenon, an interpersonal model of personal growth and well-being rooted in the social psychological literature (e.g., Drigotas et al., 1999; Rusbult et al., 2009a), to consumer–brand relationships. Drawing from the consumer–brand relationship literature, we contend that a product or brand can assume the symbolic role of a close relationship partner by eliciting aspects of consumers’ ideal selves. We present a model of how consumers can experience personal growth via *product affirmation of the ideal self* (*product affirmation* for short), or a product’s ability to elicit the consumer’s ideal self. This, in turn, is predicted to facilitate *movement toward the ideal self*, with proposed consequences for personal well-being and loyalty to the product (see Figure 1 and Table 1).

Thus, this paper contributes to both the social psychological and consumer research literature by proposing product affirmation as a mechanism through which products and brands may serve as a symbolic partner to facilitate personal growth and promote personal well-being and brand loyalty. We begin our conceptual review by describing the model of the Michelangelo phenomenon, then apply the interpersonal model to the consumer–brand relationship literature to introduce the concept of product affirmation as a mechanism for experiencing personal growth through brands. Next, we discuss preliminary evidence for the model before mapping the three theoretical foundations of the Michelangelo phenomenon (behavioral confirmation, interdependence theory, and self-discrepancy theory) onto consumer research. We then describe positive and negative consequences for personal well-being and brand loyalty and end with directions for future research and implications.

## 2. The Michelangelo Phenomenon in Close Personal Relationships

The Michelangelo phenomenon (e.g., Drigotas et al., 1999; Rusbult et al., 2009a) is an interpersonal model of personal growth and striving, which has been primarily examined in the context of romantic relationships (see Table 1 and Figure 1). The model uses the metaphor of Michelangelo Buonarroti, one of the most influential artists of all time, who allegedly described the sculpting process as chipping away the outer layers to unearth the ideal form within the stone (Gombrich, 1995). Similarly to the stone, people are also often in need of a skilled partner to help draw out their ideal self, which is a possible future self that individuals ideally desire for themselves (Higgins, 1987, 1989; Markus & Nurius, 1986). In other words, the Michelangelo phenomenon describes a ‘sculpting’ process in which close partners help shape individuals toward their ideal selves.

Key to the model (e.g., Drigotas et al., 1999; Rusbult et al., 2009a) is *partner affirmation of the ideal self*^2^, or partners perceiving and behaving in a manner that aligns with the individual’s own ideal self. Over time, and through repeated affirming interactions that draw out desired characteristics and behaviors, individuals experience *movement toward the ideal self*, whereby individuals increasingly resemble the kind of person they ideally desire to be. For example, if Jake senses Jane’s desire to be more creative, he may suggest going to art galleries together, taking art classes, and showing off her creations to friends. Over time, as a result of Jake’s encouragement, Jane may indeed feel more creative, spending more time making art and feeling confident gifting her creations to friends.

Affirmation is a continuum ranging from disaffirmation to affirmation, with some partners affirming irrelevant characteristics or even the feared, undesired self. Such non-affirming or disaffirming interactions can result in movement away from the ideal self (Drigotas et al., 1999; Rusbult et al., 2009a). For example, if Jane instead meets James who feels that her artwork is a waste of time and that she should focus on her career and family, over time, Jane may give up on creating art in her spare time and may even convince herself that James is correct in that creating art is a waste of her time.

Although the model proposes a mediational sequence with movement toward the ideal self mediating the relationship between partner affirmation and well-being, affirmation yields positive consequences for relational quality and personal well-being, independent of perceived movement (Drigotas, 2002; Drigotas et al., 1999). Even if it may be difficult to make good progress on an important goal (e.g., to get a dream job) or to display desired traits (e.g., to be confident), having an affirming partner who supports such goal pursuits or who believes in the individual’s potential can directly enhance personal well-being and make individuals appreciate being in such an affirming relationship. On the other hand, a disaffirming or non-affirming partner has negative consequences for relational and personal well-being. Going back to the previous examples, being with affirming Jake may make Jane more satisfied with both her life and her relationship, but being with disaffirming James may make her feel that something is missing in her life and in her relationship.

Empirical studies have shown support for the basic model, using experimental, correlational, and longitudinal studies with romantic couples, using self-report, partner-report, friend-report, and observations of couples discussing an ideal-relevant goal (for a review, see Kumashiro et al., 2006). Other studies have uncovered facilitators or inhibitors of the affirmation process: for example, the Michelangelo effect is facilitated to the extent that partners are similar to the ideal self, are action- or promotion-oriented, and show interpersonally adaptive personality traits such as being securely attached, agreeable, extraverted, and emotionally stable (Bühler et al., 2020; Gu et al., 2026; Kumashiro et al., 2007; Righetti et al., 2010; Rusbult et al., 2009b). Measures of partner affirmation typically rely on self-reported perceptions (e.g., “My partner behaves in a way that allows me to become who I most want to become”), while movement toward the ideal self is typically measured by asking individuals to report how much they have moved closer to their top most desired traits or goals or toward their overall ideal self.

Although the Michelangelo phenomenon (Drigotas et al., 1999; Kumashiro et al., 2006) has been studied primarily in romantic relationships, its underlying principles (behavioral confirmation, interdependence, and self-discrepancy processes) are not restricted to such relationships. The next section introduces our model of *product affirmation* and conceptually maps the three theoretical foundations to consumer–brand literature.

## 3. Product Affirmation: The Michelangelo Phenomenon in Consumer–Brand Relationships

Similarly to close relationship processes (Rusbult et al., 2009a), we propose that products and brands can serve as the ‘Michelangelo’ sculptor and facilitate movement toward the ideal self by symbolically affirming the consumer’s ideal self (*product affirmation*), eliciting, reinforcing and supporting traits, values, identities, and goals that consumers most desire for themselves. Over time and through repeated engagement with affirming brands, consumers are likely to perceive that they are moving closer to their ideal selves. Product affirmation and movement toward the ideal self are both predicted to enhance personal well-being and promote loyalty to the product. Figure 1 depicts our product affirmation model, and Table 1 illustrates the conceptual mapping of constructs described in the affirmation model.

As an illustrative example, going back to Nike’s 1995 “If You Let Me Play” campaign, the sporting corporation began a series of advertisements aimed at women with a message of commitment to female sports. In TV commercials, girls and young women proclaimed a series of empowering statements, such as, “If you let me play sports, I will have more self-confidence… I will suffer less depression… I will learn what it means to be strong,” concluding with the “Just Do It” slogan (Creative Review, 2012). This campaign example illustrates how a brand can affirm an ideal self centered around strength, confidence, athletic participation, and empowerment. Whether or not any single campaign produces measurable personal growth, the message positioned Nike as symbolically supporting a valued possible self and was associated with positive consumer responses (Eyada, 2020).

Moreover, we are not suggesting that brands literally perceive or intentionally respond to consumers’ ideal-relevant aspirations as a human might. However, the concept of partner affirmation in close relationships also relies primarily on self-reports of *perception* that their partners are eliciting one’s ideal self, rather than specific objective sets of intentional actions made by interaction partners (Kumashiro et al., 2006). The consumer–brand relationship literature shows that individuals often anthropomorphize brands (Aggarwal & McGill, 2007) or otherwise experience them in a similar manner to relationships (Fournier, 1998; Alvarez & Fournier, 2016). We therefore suggest that brands can function symbolically as affirming relationship partners by reflecting desired identities, characteristics, and values, shaping repeated behavioral patterns, and creating experiences consumers interpret as affirming. In fact, close partners who resemble the ideal self have been shown to be better sculptors (Rusbult et al., 2009b), and similarly, brands may make effective sculptors as consumers may be attracted to brands which reflect their ideals (Sirgy, 1982). We next review the relevant consumer–brand relationship literature to make a conceptual case for our model.

### 3.1. Product Affirmation: Preliminary Empirical Evidence

Although empirical research that directly tests the model of product affirmation remains limited, preliminary findings suggest support for the model. To our knowledge, three published empirical studies have directly applied the Michelangelo model to consumer-product relationships. We first introduced the concept of *product affirmation* in a pilot study (Coolsen & Kumashiro, 2009). Utilizing an experimental design, we pitted product affirmation effects (products that affirmed one’s ideal self-concept) against product verification/enhancement effects (products that verified one’s actual self-concept or symbolized one’s self-concept that others tend to favor). We found that product affirmation was associated with higher levels of personal growth and well-being outcomes than product verification or product enhancement. A follow-up correlational study then applied the product affirmation model to an educational setting and found that feeling affirmed by their university (i.e., the product) was associated with higher levels of positive self-concept and loyalty to the university (Coolsen et al., 2010).

Finally, in a correlational test of the Michelangelo model, Isaranon (2019) found that Facebook users who felt affirmed by Facebook reported higher self-esteem, which was partially mediated by perceived closeness to their ideal selves. Interestingly, moderate users of Facebook reported the greatest benefits compared to light or heavy users, suggesting some boundary conditions for products such as social media, which has been shown in other unrelated research to have mixed effects on users’ personal well-being (Marciano et al., 2024; Paul & Headley-Johnson, 2025). Moreover, the study was conducted in Thailand, suggesting that individuals in collectivistic countries also benefit from having their ideal selves affirmed, contrary to research findings that suggest there is more cultural emphasis on making progress toward the ideal self in individualistic societies (e.g., A. Y. Lee et al., 2000).

Thus, these preliminary studies suggest that individuals may be able to experience affirmation from brands such as universities or Facebook, with positive consequences for personal growth, personal well-being, and loyalty to the product. Nevertheless, direct evidence for the product affirmation model remains limited. The next sections consider consumer–brand research that provides conceptual, rather than direct empirical, support for the model. Such conceptual models are consistent with other theoretical extensions of interpersonal relationship models to brand contexts, which have similarly preceded rather than followed extensive direct empirical testing (e.g., Reimann & Aron, 2014).

### 3.2. Theoretical Foundations of the Michelangelo Model

The Michelangelo phenomenon is based on principles of the socially constructed self and the looking glass self (e.g., James, 1890; Cooley, 1902), which suggest that our self-concept, that is, a collection of our personal traits, values and behavioral tendencies, is continuously shaped by interpersonal experiences, as we turn to significant others to reflect our characters and identities (e.g., Drigotas et al., 1999; Kumashiro et al., 2006). In particular, the model draws on three distinct theoretical traditions in social psychology. First, the Michelangelo model is essentially a behavioral confirmation process (e.g., Darley & Fazio, 1980; Harris & Rosenthal, 1985), whereby another person’s expectations about the self can become self-fulfilling by eliciting behaviors that confirm the other’s beliefs. For example, in a classic study (Snyder et al., 1977), men who believed they were interacting with an attractive woman elicited more flirtatious behaviors over the phone compared to men who believed they were interacting with a less attractive woman, regardless of the actual attractiveness of the woman.

However, isolated instances of behaviorally confirming encounters are unlikely to result in lasting changes to the self-concept. Thus, the second theoretical tradition stems from interdependence theory (Kelley & Thibaut, 1978; Rusbult & Van Lange, 2003) in which long-term interdependence, characterized by strong interpersonal influence across a wide range of contexts, can result in more stable changes in the self-concept over time. The final theoretical tradition draws on self-discrepancy theory (Higgins, 1987, 1989) that suggests that whether such stable changes have positive or negative effects might depend on whether close partners are eliciting aspects of the self-concept aligned with the ideal self or other concepts that are irrelevant or antithetical to the ideal self. Benefits are especially likely to occur when interdependent partners in long-term relationships sculpt the self toward the self’s ideals. We now review the relevant literature in consumer branding that conceptually maps onto the close relationship literature for these three theoretical foundations.

#### 3.2.1. Behavioral Confirmation Principles

In the context of consumer–brand relationships, processes similar to behavioral confirmation principles (e.g., Darley & Fazio, 1980; Harris & Rosenthal, 1985) may occur when products or brands evoke a specific social identity, triggering a set of internalized or social expectations and norms that consumers then act upon. Consumer identity research suggests that brands can serve as symbolic resources for constructing and expressing the self (e.g., Escalas & Bettman, 2005; Saint Clair, 2023). For example, identity-based motivation (Oyserman, 2009) shows that when an identity is salient, individuals adopt an identity-congruent mindset in understanding and interpreting the world around them and become motivated to pursue goals and behaviors congruent with their identity. Symbolic consumption literature also suggests that consumers use symbolic brands not only to communicate an existing identity but also to construct and strengthen their self-concept, drawing meanings from reference groups and culturally shared associations (e.g., Belk, 1988; Escalas & Bettman, 2005). Research on narrative identity also suggests that narrative processing can strengthen self-brand connections by enabling consumers to integrate brand meanings with autobiographical memories and stories about who they are (e.g., Escalas, 2004).

For example, ecologically minded consumers may engage in more identity-congruent behaviors when engaging with brands like Patagonia, known for its environmental activism stance, by paying a premium for sustainably sourced products, recycling, and joining community groups that advocate such efforts (Singh et al., 2022). Ethnic-based brand representations (e.g., a hijab-wearing Barbie doll) have been shown to strengthen social identification, allowing consumers to see their heritage reflected and validated in the marketplace (Mishra & Bakry, 2021).

Moreover, behavioral confirmation processes may also operate through brand communities, or social networks of like-minded consumers who expect members to enact behaviors associated with the brand, offering incentives for remaining in the group (e.g., Muniz & O’Guinn, 2001). For example, research findings show that brands on social media nurture the needs and aspirations of their consumers, including their passions and feelings of connection and self-expression (Santos et al., 2022). Luxury items were shown to provide consumers with enhanced social value and group acceptance, as well as positive emotional experiences, such as happiness and well-being (Xi et al., 2022). Thus, the first theoretical foundation process of behavioral confirmation may occur in brand contexts through attempts to meet both internal and external expectations of brand norms and social expectations.

#### 3.2.2. Interdependence Theory and Brands as Relationship Partners

The second theoretical foundation, interdependence theory (Kelley & Thibaut, 1978; Rusbult & Van Lange, 2003), addresses why having a long-term interdependent partner is important for lasting changes. It may be questioned whether products or brands can be compared to an interdependent partner, capable of shaping the self-concept over time. Research on consumer–brand relationship theory (Fournier, 1998) established that consumers often regard brands as a meaningful relationship partner above and beyond the simple utilitarian function that products serve in the marketplace. For example, consumers have been shown to react negatively when they perceive that brands violate expectations of communal relationships (Aggarwal, 2004), suggesting that consumers have expectations of brands showing care and being responsive to their needs. A study of Patagonia customers found that consumers develop a consumer–brand relationship that demonstrates characteristics such as partner quality, love, passion, and interdependence (Singh et al., 2022).

Fournier (1998) provided case-based evidence of the various relationship forms (e.g., arranged marriages, casual friends/buddies, committed partnership) that consumers can take on with the brands in their lives. Consumers attribute human-like characteristics to brands, form emotional attachments, express love and commitment, and include brands in their self-concept. As a result, relationships with brands mirror interpersonal relational dynamics, with similar relational outcomes/pro-relationship behaviors such as forgiveness, partner attribution biases, and derogation of alternatives (Alvarez & Fournier, 2016; Fournier, 1998).

Moreover, consumers can develop dependence on brands in a comparable manner to relationship partners. For example, research applying Rusbult’s (1983) investment model of commitment to relationship partners found that brand commitment is similarly predicted by high levels of satisfaction and investment (e.g., time and resources put into the brand) and low quality of alternatives. Some participants who were not satisfied nevertheless were committed to the brand to the extent they reported high investment, along with perceived lack of alternatives (Sung & Choi, 2010).

For example, some essential utilities like smartphones (e.g., Apple vs. Android) may make consumers more dependent on a particular brand since switching between the two operating systems across a variety of apps and devices may be more difficult, making consumers invest more and perceive that other brands are not credible alternatives. An academic case study of Apple’s brand community found markers of religiosity, such as shared beliefs, ritual behavior, and reverence for the brand, that persisted even after the specific product, the Apple Newton, was discontinued by the company (Muniz & Schau, 2005), suggesting that consumers’ devotion to a brand can become decoupled from any single product’s ongoing utility.

Thus, repeated meaningful interactions with products and brands may create psychological interdependence similar to a close relationship, with consumers relying on brands to support important goals, express identities, reinforce values, and maintain everyday activities.

#### 3.2.3. Self-Discrepancy Theory

Finally, the third theoretical foundation of the Michelangelo phenomenon, self-discrepancy theory (Higgins, 1987, 1989), has some parallels in the consumer–brand relationship literature. Early consumer research on the association of brands with the self-concept focused on brands and products as extensions of the self, such as through nurturing and customizing automobiles as a direct function of their ego ideals (Belk, 1988). Consumers may stop using and/or dispose of products when they cease to reflect their ideal self-image.

Self-image congruity theory (Sirgy, 1982) posits that consumers are drawn to purchase brands that reflect aspects of their self-concepts. Relevant to our product affirmation model, ideal congruity (similarity between products and consumers’ ideal self) has been robustly associated with consumer choice in the forms of product preference, usage, loyalty, and ownership. Early research (Malhotra, 1988) found that there were higher preferences for house profiles that were congruent with one’s ideal self compared to profiles congruent with one’s actual self (actual congruity). More recently, research has found evidence for this ideal congruity effect in both retail and sporting event environments (Ibrahim & Najjar, 2008; Moharana et al., 2023). Furthermore, consumers perceived greater authenticity in brands that were congruent with their ideal self-concepts, compared to brands congruent with their actual self-concepts (Zogaj et al., 2024).

Nevertheless, research on self-image congruity also shows that ideal and actual self-congruity can exert different effects. Across a wide range of 167 brands, congruence with the actual self, rather than with the ideal self, was associated with stronger emotional brand attachment, especially for individuals with high self-esteem, low public self-consciousness, and higher involvement with the product (Malär et al., 2011). Therefore, there may be important boundary contexts in which alignment with the actual or ideal self is more important.

This is consistent with theorizing of the Michelangelo model, which suggests that affirmation of the ideal self and verification of the actual self can work in tandem. Just as a skilled sculptor needs to be aware of how best to chisel away the outer layers to unearth the ideal self, the model theorizes that partners who are aware of the actual self, including potential flaws and weaknesses, may be better positioned to elicit the ideal self, acknowledging the actual self while bringing out the ideal aspects of the self (e.g., Kumashiro et al., 2006; Rusbult et al., 2009a). Similarly, in the case of product affirmation, consumers may alternate between brands that elicit the actual and the ideal selves. Given that self-esteem is often conceptualized as low discrepancy between the actual and ideal selves (e.g., Rogers, 1961; James, 1890), Malär et al.’s (2011) findings might also suggest that high self-esteem consumers with low public self-consciousness, who already feel close to their ideal selves and may not be seeking public validation, may be more likely to stick to a brand that is able to reflect both the actual and ideal selves, rather than switch to more popular brands that may reflect a more unrealistic ideal image.

Symbolic self-completion theory (Wicklund & Gollwitzer, 1981) may also help explain the mixed findings for preference for brands that match actual or ideal images. According to this theory, individuals purchase certain products as a means of symbolically closing a felt gap between their actual and desired identity, particularly following a self-threat. For example, compulsive buying has been interpreted as an attempt to symbolically move closer to one’s ideal self (Dittmar, 2005). This account offers one explanation for why, under some conditions, consumers may gravitate toward brands that signal an aspired-to identity rather than one that simply reflects the actual self.

Nevertheless, the Michelangelo phenomenon (e.g., Rusbult et al., 2009a) describes a dynamic process of personal growth that is not contingent on a prior self-threat, and it remains unclear whether pursuing a product for symbolic self-completion results in genuine movement toward one’s ideal self or whether the resulting sense of closure remains merely symbolic. The product affirmation model also differs from other theories that describe self-restorative processes triggered by an antecedent threat, such as self-affirmation theory (Steele, 1988). Self-affirmation theory posits that reflecting on a valued, self-defining domain can restore self-integrity following a threat, which can even reduce preference for products that might otherwise serve a compensatory function (Townsend & Sood, 2012). We suggest that these threat-based theories may be useful to examine as complementary theories or moderating factors in future research.

It is also important to note that our model of product affirmation differs from, and extends, self-congruity theory in important ways. While self-congruity theory (Sirgy, 1982) focuses on the static fit between a consumer’s self-concept and brand image, our product affirmation model proposes a dynamic model of personal growth, with brands symbolically serving as an interdependent partner (e.g., Fournier, 1998), helping consumers move toward their ideal selves through repeated interactions that elicit the ideal self.

Thus, our discussion so far describing the limited empirical evidence on product affirmation and our conceptual mapping of the three theoretical foundations of the Michelangelo model (behavioral confirmation, interdependence, and self-discrepancy) to the consumer–brand relationship literature suggest that products and brands may be able to affirm the consumer’s ideal self and move them closer to their ideal self (see Figure 1), in much the same manner as a close relationship partner. Our extended model (see Figure 1) also includes consequences for personal and relational well-being (e.g., brand loyalty), which we describe next.

## 4. Product Affirmation Consequences: Personal Well-Being and Brand Loyalty

As described earlier, research on the Michelangelo model (e.g., Drigotas et al., 1999; Drigotas, 2002) has demonstrated that both partner affirmation and movement toward the ideal self promote relational and personal well-being. Empirical research shows that partner affirmation may sometimes exert stronger effects on well-being than actual movement, especially for personal well-being, given the difficulty in making actual progress toward ideal-relevant goals or traits (Bühler et al., 2019, 2020; Drigotas et al., 1999; Drigotas, 2002). The model proposes that analogous processes may occur with brands, with product affirmation expected to promote brand loyalty and personal well-being (see Figure 1). Although direct empirical evidence for the consequences of the product affirmation model remains limited, we describe some consumer research findings that show indirect support for the model.

The consumer–brand relationship literature shows that brands inspire loyalty and enhance personal well-being through providing substantial meaning in a consumer’s life, beyond merely satisfying functional needs. Brands influence well-being across multiple dimensions, depending on consumer characteristics and context. For example, brands can elevate positive affect: prestigious brands can serve to increase happiness and well-being (Kumar et al., 2021), while sustainability-minded consumers experience a “warm glow,” stemming from moral well-being, when enacting constructive behavior toward the environment via green and sustainable branded consumption (Choi & Ahn, 2023).

Brands can facilitate consumer engagement and strengthen self-brand connections, especially when brand communications are perceived as being authentic (Kautish et al., 2025). This can occur via varied pathways: for example, meaningful in-store experiences or a value-expressive brand’s packaging can strengthen a consumer’s emotional bond with brands and can bolster well-being (Neacșu, 2025; Sciarrino & Prudente, 2025). Watching branded videos was found to be associated with the “personal growth effect” by inspiring and motivating consumers and providing meaning (Chang, 2020). Even in times of distress, brands may be experienced as providing a supportive environment to mothers grieving the loss of their infant children (Colligan et al., 2025). Thus, similar to the Michelangelo process (Drigotas et al., 1999; Drigotas, 2002), the consumer–brand relationship literature suggests that brands may be able to promote personal and relational well-being of consumers via paths similar to affirmation and movement toward the ideal self.

## 5. Negative Consequences

In the Michelangelo model (Drigotas et al., 1999; Rusbult et al., 2009a), partner affirmation is conceptualized as a continuum, ranging from disaffirmation and non-affirmation to affirmation of the ideal self, with positive consequences when partners affirm the ideal self. We propose that products and brands that are perceived to elicit irrelevant aspects of the self, actively disaffirm the self, or impose their vision of the ideal self or ideal life onto consumers will be associated with negative consequences (see Table 1). For example, research on the Michelangelo phenomenon identified a related construct termed the Pygmalion effect (see Kumashiro et al., 2006), based on Shaw’s (1941) play, *Pygmalion*, which depicts negative consequences for the “sculptor,” Higgins, when he attempts to mold the working-class flower seller, Eliza, into his vision of the ideal woman, a lady, but fails to support her ideal aspirations and identity goals after the transformation.

In the brand context, an illustrative example of the Pygmalion effect and its relationship to imposed affirmation can be seen in the narrative of the film *The Devil Wears Prada* (Frankel, 2006). Here, the luxury fashion environment is portrayed as imposing an external identity standard onto the protagonist, Andy (e.g., the consumer). Although Andy undergoes a successful outward transformation, this external molding seems to operate as an impeding pathway that moves her away from her intrinsic ideal self of becoming a serious journalist, with negative consequences for her personal and relational well-being. This dynamic culminates in brand rejection, as Andy eventually quits her job and discards her luxury wardrobe. 

Thus, even when a transformative story may be considered to be objectively successful (e.g., a poor, uneducated street flower seller becoming a proper “lady” in *Pygmalion* or becoming fashionable and successful in *The Devil Wears Prada*), the consequences may not yield lasting benefits if such external metrics of success do not align with the individual’s own ideal self. Interestingly, empirical work in consumer psychology also suggests that explicit identity-marketing messages that directly link a brand to a consumer’s desired identity can backfire and reduce purchase likelihood when consumers perceive a threat to their sense of agency in choosing and expressing that identity themselves (Bhattacharjee et al., 2014). Even when an imposed identity is otherwise a good objective fit, consumers may reject a brand that leaves them feeling less like the author of their own transformation and more like its target.

Moreover, research on brand congruity (Sirgy, 1982) also shows that consumers avoid brands that are considered to be incongruent with their desired identity, particularly brands that elicit the undesired self (M. S. Lee et al., 2009; Mostert & Naude, 2022). Such incongruity effects are analogous to perceptions of being disaffirmed by the brand. Brands may even risk alienating consumers if they make changes that result in incongruity with their valued identity. For example, Sayin and Gürhan-Canlı (2024) found that when a brand’s actions became symbolically incongruent with its own established meaning, consumers with high self-brand connection, that is, those most likely to have incorporated the brand into their identity, reported greater feelings of betrayal alongside declining brand attitudes, reduced purchase intentions, and stronger intentions to engage in negative word of mouth. Notably, this reaction occurred even without any unethical or unfair conduct on the brand’s part, driving a perceived shift in what the brand symbolically represented. 

Furthermore, brands may also affirm maladaptive or unrealistic ideal selves, with associated negative consequences. Advertisements often depict unhealthy beauty standards or encourage consumers to pursue their brands for extrinsic reasons (e.g., to gain wealth, admiration, prestige, and status). Research on self-determination theory shows that pursuing a goal for intrinsic motivation leads to personal growth and higher levels of well-being, compared to extrinsically motivated pursuits (e.g., Deci & Ryan, 2000; Kasser & Ryan, 1993). As such, consumers who pursue brands for extrinsic reasons or due to external pressures are less likely to move closer to their ideal selves or experience greater well-being. For example, advertisements showing idealized and unrealistic body image may lower self-esteem and body satisfaction and widen perceived discrepancy between the actual and ideal self (for review, see Consiglio & van Osselaer, 2022). Luxury brands may also lead to “imposter syndrome” or feelings of inauthenticity, leading consumers to behave less confidently when engaging with their products (Goor et al., 2020). 

As consumers actively seek out brands to elicit desired identities, brands risk inadvertently affirming extrinsically motivated desires or maladaptive, unrealistic ideal selves. Such risk may be exacerbated by the recent introduction and the rapid proliferation of Large Language Model (LLM) based AI systems, which are being used by both consumers in their search for products and by corporations to target direct advertising (e.g., for reviews, see Chintalapati & Pandey, 2022; Sujood et al., 2026; Ribeiro et al., 2025). Because generative conversational LLMs optimize for continuous user engagement and positive feedback ratings, they may be highly susceptible to ‘AI sycophancy’—an algorithmic tendency to uncritically flatter, praise, or validate a user’s stated worldview regardless of its objective accuracy or psychological health (Sharma et al., 2024). In digital marketing contexts, automated systems risk over-affirming a consumer’s unrealistic ideal self to maximize platform interaction metrics. 

A related risk is generative AI’s tendency to hallucinate, or generate plausible but false content (Grewal et al., 2025), which could lead an LLM-powered marketing system to fabricate an unfounded personalized claim about how well a brand fits a consumer’s identity or goals, lending false confidence to an imposed or extrinsically motivated ideal self. While a comprehensive analysis of this technology is outside the scope of this paper, its capacity to instantly synthesize consumer data to generate personalized marketing content and communication (Grewal et al., 2025), combined with its responsive, conversational interface that drives active customer engagement (Polimerou & Spais, 2025), may encourage some consumers to view AI-driven brands as active relationship partners. Whether these automated affirmation processes facilitate actual personal growth or drive negative consumer outcomes is an important question for future research.

Thus, to experience personal growth and well-being, it is important that brands avoid imposing their ideal standards and instead affirm individuals’ own ideal selves and that consumers reflect on whether they are pursuing a brand to meet their intrinsic needs or for extrinsic purposes. The same behavioral confirmation and interdependence principles of the Michelangelo phenomenon (e.g., Kumashiro et al., 2006; Rusbult et al., 2009a) apply when brands elicit undesired or extrinsically motivated aspects of the self; in such cases, consumers may gradually move away from their ideal selves over time. Nevertheless, similar to the protagonists’ journeys in *Pygmalion* and *The Devil Wears Prada* (Frankel, 2006; Shaw, 1941), it may take time before consumers realize that such sculpting processes by brands may ultimately be impeding their personal growth strivings and well-being (Kumashiro et al., 2006; Rusbult et al., 2009a).

## 6. Implications and Future Directions

The product affirmation model offers several theoretical and practical contributions. In drawing on separate literature on self-concept, close personal relationships, and consumer–brand relationships, we offer a conceptual mapping of how the Michelangelo phenomenon (Drigotas et al., 1999; Rusbult et al., 2009a), an interpersonal model of personal growth, can apply to brand contexts.

### 6.1. Theoretical Implications

The present conceptual review extends both the consumer–brand and close relationships literature in important ways. Building on the long-standing notions of the socially constructed self and the looking glass self (e.g., James, 1890; Cooley, 1902), we suggest that brands may also be able to help shape consumers’ self-concepts and identities. Beyond their functionally intended uses, brands can serve as long-term meaningful interdependent partners who repeatedly reinforce and support consumers’ desired aspirations, values, characteristics, and identities. Our model thus contributes to the consumer–brand relationship literature (Alvarez & Fournier, 2016; Fournier, 1998) by specifying new psychological mechanisms (product affirmation and movement toward the ideal self), through which consumers can attribute human-like characteristics to brands and strengthen their attachment and loyalty to the brand.

Moreover, our model differs from existing consumer–brand theories such as self-congruity theory (e.g., Sirgy, 1982) and symbolic self-completion theory (Wicklund & Gollwitzer, 1981). Unlike self-congruity theory, which focuses on static congruence between the ideal self and brand image, and symbolic self-completion theory, which concerns symbolic closure of an identity gap, our model illustrates a dynamic process whereby repeated affirming interactions with a brand may yield movement toward the ideal self and help promote personal well-being and brand loyalty. In addition, our model also contributes to other identity- and group-based consumer–brand relationship literature (e.g., Muniz & O’Guinn, 2001; Oyserman, 2009; Saint Clair, 2023), suggesting further implications when a consumer’s identification with the brand and like-minded fellow consumers are in alignment with the ideal self.

Furthermore, our model of product affirmation contributes to the close relationship literature. The Michelangelo phenomenon (Drigotas et al., 1999; Rusbult et al., 2009a) has primarily been examined in the context of romantic relationships, though its underlying principles suggest that the model can apply to other relational contexts. In this review, we outline how similar affirmation processes may occur symbolically through repeated engagement with affirming brands. Already, self-expansion theory (Aron & Tomlinson, 2026), which posits that individuals experience personal growth through their partner by incorporating aspects of the other into the self and engaging in novel, self-expanding activities, has been applied in the context of brands (Reimann & Aron, 2014). This work may thus contribute to the emerging literature in close relationships on how individuals can thrive through their relationships (Feeney & Collins, 2015), where brands may also help contribute to personal growth processes.

Nevertheless, as noted previously, we are not suggesting that brands are literally perceiving and intentionally responding to the consumers’ ideal selves in a similar manner to an affirming partner. Rather, we have illustrated how brands serve to symbolically affirm the ideal self, consistent with the broader tradition of research on brand anthropomorphism (Aggarwal & McGill, 2007; Alvarez & Fournier, 2016; Fournier, 1998) and extending the self through brands (e.g., Belk, 1988).

### 6.2. Practical Implications for Marketers

Our product affirmation model carries implications for branded strategy. From a managerial perspective, the product affirmation model suggests that brands should not be evaluated only by functional utility or symbolic congruence, but also by the extent to which they support consumers’ intrinsically valued movement toward ideal selves. The key shift is moving from “What problem does this product solve?” to “What identity does this brand help the consumer express or sustain?” A brand might support an ideal self centered around being healthier, more creative, more responsible, or more connected. Effective positioning would then connect branded products or services to those aspirations in ways that resemble a responsive, supporting partner, helping individuals move closer to their ideal selves.

Brand loyalty looks different through this framework. Consumers may stay loyal not just because a brand performs well, but because it helps them maintain a desired sense of self. Loyalty programs can reflect this by moving beyond points and discounts toward rewards that support activities that will help consumers move closer to their ideal self, including opportunities for skill-building, community recognition, learning experiences, or meaningful access. Brands that have taken this approach, as Patagonia did around environmental activism, have been shown to retain a loyal following (Singh et al., 2022). Such repeated opportunities to enact environmentally responsible behavior in the presence of like-minded community members may reflect all three tenets of the product affirmation model (behavioral confirmation, interdependence, and self-discrepancy processes).

The framework also cautions marketers against positioning brands as Pygmalion-like partners that impose a brand-defined ideal self and promote unrealistic or maladaptive ideals. Brands should avoid prescribing what consumers ought to become or linking identity membership too directly to purchasing behavior. Explicit identity-marketing messages can reduce consumer agency and produce resistance even when the identity itself is valued (Bhattacharjee et al., 2014). Managers should therefore consider not only engagement and loyalty but also whether consumers experience the brand as supporting autonomy, sustainable ideal-relevant behavior, and personal well-being. Conversely, perceptions of pressure, inauthenticity, unrealistic standards, or movement toward an externally imposed identity may indicate disaffirmation or imposed affirmation. Given the limited direct evidence for the complete product affirmation model, these recommendations should be regarded as theory-derived principles requiring further empirical evaluation.

### 6.3. Future Directions

Although preliminary empirical findings provide initial support for the product affirmation framework, direct empirical research remains limited, with just three preliminary studies (Coolsen & Kumashiro, 2009; Coolsen et al., 2010; Isaranon, 2019). We note that our model’s proposed sequence (see Figure 1, from product affirmation, to movement toward the ideal self, to enhanced well-being and brand loyalty) reflects a theorized rather than an empirically established causal pathway. While longitudinal and experimental evidence supports this sequence in the original interpersonal Michelangelo phenomenon (e.g., Kumashiro et al., 2006), more research is needed to test the validity of the model, using rigorous, experimental methods and employing longitudinal designs to test long-term impacts of the affirmation model. Future research should also develop and validate measures of product affirmation and movement toward the ideal self, for example, by adapting existing partner-affirmation items to product or brand contexts and assessing perceived progress toward self-nominated ideal traits or goals, or change in actual–ideal self-discrepancy over time. More research is also needed to examine antecedents, mechanisms, moderators, and long-term consequences of our product affirmation model. Next, we outline several promising avenues for future research.

First, research on the Michelangelo phenomenon suggests that dispositional traits may affect who will benefit from product affirmation. Traits such as being action- or promotion-oriented, securely attached, agreeable, extraverted, and emotionally stable have all been shown to elicit more affirming behaviors from romantic partners (Bühler et al., 2020; Gu et al., 2026; Kumashiro et al., 2007; Righetti et al., 2010; Rusbult et al., 2009a). Affirmation may also play a moderating role: narcissistic individuals were more likely to remain committed to their romantic relationships to the extent that their partners affirmed their ideal selves (Gloor et al., 2025). Age was not found to influence the Michelangelo process (Bühler et al., 2019), but in the context of brands, it is possible that age may affect which products and advertising campaigns individuals are drawn toward. Given that Isaranon (2019) found evidence for product affirmation in collectivistic cultures, future research should also examine whether independent/interdependent self-construals may matter in pursuit of the ideal self (A. Y. Lee et al., 2000).

Future research should examine these and other dispositional traits to see if they influence the product affirmation process, and if such traits influence brand relationships in the same way as close relationships. For example, research already suggests that promotion-oriented individuals may be better persuaded by messages that appeal to their eager strategies, whereas prevention-oriented individuals respond better to vigilant strategies (A. Y. Lee & Higgins, 2009), and as such, the fit between message framing and dispositional traits may also matter in the product affirmation process. Future research might also examine individual differences in self-regulatory capacity, such as trait self-control and susceptibility to self-regulatory depletion, as a moderator of consumers’ ability to sustain movement toward the ideal self through repeated brand engagement, given that goal pursuit over time draws on the same regulatory resources studied in the broader consumer self-regulation literature (Francke & Carrete, 2023). Other consumer–brand relationship research suggests that dispositional traits often affect brand relationships in a different way from close relationships, such as consumers with insecure attachment styles using brands to compensate for interpersonal relationship deficiencies or as a signaling device to help improve their close relationships (e.g., Alvarez & Fournier, 2016).

Moreover, future research should examine other potential mechanisms and moderators. For example, not everyone is clear about the kind of person they wish to become. Individuals with a clear and stable sense of their self-concept (self-concept clarity; Campbell et al., 1996) may be in a better position to recognize which brands are affirming their ideal selves, whereas those who do not know what they want may be turning to brands to help them define their identity. Research has already shown that consumers with low self-concept clarity tend to use brands as anchors of their self-concept identity (Mittal, 2015). Related research on self-expansion theory applied to brands (e.g., Reimann & Aron, 2014) suggests that individuals are motivated to expand their self-concepts by incorporating brands, leading to acquisition of new perspectives, resources, and identities just as a close partner might do for them. Brands that enable consumers to develop new skills, explore new identities, or participate in new communities may facilitate product affirmation and enable them to feel closer to their ideal selves. Thus, future research could examine clarity of a consumer’s ideal self and the extent to which self-expansion processes may affect the product affirmation process.

Finally, future research should also examine negative aspects more closely, as there may be different risks than a human partner. As discussed (see Table 1), the dark side of product affirmation can take at least two forms: (1) disaffirmation, in which a brand evokes an undesired or irrelevant self, and (2) imposed affirmation, in which a brand affirms an ideal that is extrinsic, unrealistic, or maladaptive, or a brand functions like a Pygmalion figure by substituting its own vision of the ideal consumer for the consumer’s intrinsically valued ideal self. Research on such processes is likely to become more important, as new and powerful AI systems, as discussed previously, may make it more likely that consumers will start experiencing brands as a responsive interdependent partner, with implications for personal growth, personal well-being, and brand loyalty.

## 7. Conclusions

This conceptual review paper proposes a model of product affirmation, a dynamic model of product-facilitated personal growth, as a conceptual bridge between research on close relationships and consumer–brand relationships. Drawing on the Michelangelo phenomenon (e.g., Rusbult et al., 2009a) and consumer–brand relationship literature (e.g., Fournier, 1998), we argue that consumers may experience brands as symbolic relationship partners that can affirm consumers’ desired possible selves, support perceived movement toward those ideal selves, and generate consequences for both personal well-being and brand loyalty. We describe preliminary empirical evidence, and we conceptually map consumer relationships research onto the three theoretical tenets of the original Michelangelo model (behavioral confirmation, interdependence, and self-discrepancy theories). The current paper thus extends both close relationships and consumer–brand relationship theories by identifying affirmation as a mechanism through which brands may become meaningful in consumers’ lives. At the same time, the model raises important ethical and empirical questions. Brands may affirm consumers’ intrinsically valued aspirations, but they may also disaffirm and impose ideals that undermine personal growth, personal well-being, and brand loyalty. Future research should therefore examine not only whether brands help consumers become who they want to be, but also when such influence supports authentic personal growth rather than merely strengthening consumer attachment.

## Figures and Tables

**Table 1:** Conceptual mapping of the Michelangelo phenomenon to product affirmation.

Michelangelo Phenomenon Construct	Close Relationship Meaning	Product Affirmation Parallel
Partner affirmation	Partner perceives and behaves in ways that align with the individual’s ideal self	Product reflects, reinforces, or elicits the consumer’s desired identity, values, goals, or aspirations
Movement toward ideal self	Individual comes to feel or act more like the person they ideally want to become	Consumer comes to feel or act more like the person they ideally want to become
Relationship well-being	Greater satisfaction, commitment, relational quality, attachment, and pro-relationship behaviors (e.g., accommodation, forgiveness, willingness to sacrifice)	Greater product satisfaction, loyalty, attachment, and pro-product behaviors (e.g., advocacy, forgiveness, or resistance to alternatives)
Personal well-being	Greater self-esteem, purpose, mental health, or life satisfaction	Greater self-esteem, purpose, mental health, or life satisfaction
Disaffirmation	Partner elicits irrelevant, feared, or undesired selves	Product reflects, reinforces, or elicits the consumer’s identity, values, goals, or aspirations that are irrelevant to the ideal self or are part of feared or undesired selves
Imposed affirmation	Pygmalion-like effect, where the sculptor imposes their own vision rather than supporting the person’s ideal self	Product imposes aspirational identities that reflect external status metrics or unrealistic standards, rather than eliciting the consumer’s intrinsic ideal self

## Data Availability

No new data were created or analyzed in this study.

## Notes

1 We use the term product affirmation to remain consistent with the original formulation of the construct and the early empirical studies that applied the Michelangelo phenomenon to consumer contexts. In this paper, we use “product” broadly as an umbrella term encompassing tangible goods, brands, services, institutions, platforms, and branded experiences. We mainly focus on brands in this paper as brands are more likely to be perceived as serving as symbolic partners, and the majority of the research we review is from consumer–brand relationships.

2 Earlier studies on the Michelangelo phenomenon distinguished between perceptual (perceiving) and behavioral (behaving) components of partner affirmation of the ideal self (e.g., Drigotas et al., 1999). Due to high statistical overlap between the two constructs, later research (e.g., Kumashiro et al., 2007) has examined partner affirmation as a combined construct. We adopt this latter convention, especially as products and brands are more likely to be able to elicit behavioral components of the ideal self.
